# Implementation of Metafounders in the Genomic Prediction for the Polled Trait in Brahman Cattle Using a Multi‐Breed Population

**DOI:** 10.1002/age.70211

**Published:** 2026-09-29

**Authors:** Lanna Maryana Costa Pereira, Fernando Baldi, Ludmilla Costa Brunes, Luís Camaripano, Cláudio Ulhôa Magnabosco

**Affiliations:** ^1^ Federal University of Goiás Goiânia Goiás Brazil; ^2^ School of Animal Science and Food Engineering University of São Paulo Pirassununga São Paulo Brazil; ^3^ Grupo Ganadero Estancias Espíritu Santa Cruz de la Sierra Bolivia; ^4^ Embrapa Cerrados Brasília Brazil

**Keywords:** Brahman cattle, genomic selection, horn development, metafounders, multi‐breed population, ssGBLUP

## Abstract

The absence of horns (polled trait) in cattle is associated with better animal welfare by reducing the need for dehorning. In Brahman cattle, population size and the scarcity of phenotypic records challenge the accuracy of genomic predictions for this trait. This study aimed to evaluate the genetic architecture of this trait in Brahman cattle using single‐breed and multi‐breed genomic prediction models, including metafounders, and to assess the influence of phenotypic classifications on heritability, prediction accuracy, bias, and dispersion. Phenotypic and genotypic data from 14 333 Nelore and Brahman animals were analyzed using threshold and linear models implemented in the BLUPF90+ software suite. Single‐breed models (*n* = 12) were considered phenotypes (polled, scab, scur, horned), while multi‐breed models (*n* = 10) incorporated metafounders and breed effects. Variance components were estimated via threshold models with Gibbs sampling, and predictive ability was assessed using the LR method with datasets. Single‐breed models showed heritability estimates (*h* ^2^) ranging from 0.56 to 0.72, but with predictive ability reflecting the Brahman population size. Multi‐breed models improved predictive performance. Results were obtained with Model 20, which included alternative phenotypes, metafounders, and breed effects, yielding accuracy (0.79), reduced bias (0.36), and dispersion close to 1. These findings demonstrate that the polled trait in Brahman cattle exhibits additive genetic variability amenable to selection. The use of multi‐breed analyses and metafounders enhances predictive accuracy, providing a strategy for populations and traits, and enables the inclusion of traits as genomic selection criterion to increase allele frequency in Brahman cattle and improve animal welfare.

## 1 Introduction

Traits associated with productive and economic performance in indicine cattle have traditionally been the focus of research on beef cattle. However, another trait that has recently gained prominence, due to its strong association with improved management conditions and animal welfare, is the absence of horns, also referred to as the polled phenotype. This condition provides benefits such as increased safety for operators and reduced damage to hides and carcasses (Youngers et al. 2017; Stafuzza et al. 2018; Gehrke et al. 2020).

In line with current animal welfare guidelines aimed at minimizing invasive and traumatic management procedures (Serighelli Júnior et al. 2022), the selection of naturally polled animals has emerged as a promising alternative. Since the early 20th century, studies have sought to elucidate the genetic and biological mechanisms underlying horn development in cattle (Bateson and Saunders 1902; White and Ibsen 1936). These early reports suggested that the phenotype was influenced by a single gene.

Subsequent research, however, has demonstrated that the genetic basis of this trait is more complex than initially described (Gehrke et al. 2020). Four intergenic variants associated with the polled trait have been identified: the “Celtic” and “Friesian” variants, found in British Scandinavian and Dutch Friesian cattle, respectively (Medugorac et al. 2012; Rothammer et al. 2014), as well as the Mongolian (PM) variant, originating from Yak and Turano cattle, and the Guarani (PG) variant, identified in Nelore cattle ( *Bos indicus* ) (Medugorac et al. 2017; Utsunomiya et al. 2019).

For indicine breeds, the genetic basis of the polled trait remains incompletely understood (Stafuzza et al. 2018), and numerous studies have aimed to identify causal genes or molecular mechanisms associated with the trait (Mariasegaram et al. 2012; Utsunomiya et al. 2016; Stafuzza et al. 2018). In addition to the standard horned and polled phenotypes, cattle may also exhibit scurs and scabs (Asai et al. 2004). These structures resemble horns but are not attached to the cranial frontal bone (Medugorac et al. 2012), ranging from small vestiges to rudimentary horn tissue up to 15 cm in length (Capitan et al. 2011).

According to Gehrke et al. (2020), sex strongly influences the expression of horn phenotypes, with horned, scurred, and scabbed phenotypes being more frequently observed in males. A recent study by Temp et al. (2024) on Nelore cattle demonstrated that genomic prediction models accounting for non‐autosomal chromosomes and four phenotypic categories—polled, polled from horned sire, scurs/scabs, and horned—achieved improved predictive ability and reduced bias and dispersion. These findings highlight the importance of sex and precise phenotypic classification for enhancing the genomic prediction of the polled trait.

In Brahman cattle, which have a relatively small population size compared with Nelore cattle in Brazil, the reliability of genomic predictions is often limited, particularly for traits influenced by multiple factors and for which phenotypic records are scarce (Londoño‐Gil et al. 2024). One strategy proposed to overcome these limitations is to combine data from multiple breeds to establish a multi‐breed reference population, thereby increasing prediction accuracy (Calus et al. 2018; Steyn et al. 2019; Ma et al. 2024; Zhang et al. 2024).

Given that the Nelore cattle have contributed to the genetic background of Brahman cattle (Koufariotis et al. 2018), combined genomic predictions for the polled trait that incorporate both populations may be effective. In scenarios where different breeds are analyzed together and pedigree information is scarce (Junqueira et al. 2020), the use of metafounders has been proposed as a solution. Porto‐Neto et al. (2013) demonstrated that distinct cattle populations often share common founders. Metafounders, described as pseudo‐individuals that preserve genetic relationships within the base population (Legarra et al. 2015), enable the genetic connection between individuals from different breeds. Their application has been shown to enhance compatibility between pedigree and genomic information (Kluska et al. 2018; Meyer and Swan 2019).

Considering the likely polygenic architecture of the polled trait (Grobler et al. 2021), the single‐step genomic best linear unbiased predictor (ssGBLUP; Legarra et al. 2009) emerges as a valuable tool, as it allows the simultaneous inclusion of genotyped and non‐genotyped animals in the analysis (Masuda et al. 2022). This method enables estimation of marker effects across the genome and prediction of genomic breeding values for the trait (Temp et al. 2024).

Therefore, using a multi‐breed population composed of Nelore and Brahman cattle, combined with metafounders that account for the shared ancestry between breeds, may constitute an effective strategy to improve the genomic prediction of the polled trait in Brahman cattle. Accordingly, the aims of this study were to perform the genomic prediction of the polled trait in Brahman cattle, to identify appropriate phenotypic classifications for this breed, and to evaluate the effects on prediction accuracy, bias, dispersion, and heritability estimates using a multi‐breed population that included metafounders.

## 2 Materials and Methods

### 2.1 Phenotypic Dataset

The data used in this study are part of databases of the Genetic Improvement Programs for Nelore (PMGRN) and Brahman (PMGRB) cattle, coordinated by the National Association of Breeders and Researchers (ANCP, Ribeirão Preto, Brazil).

Phenotypic and genotypic records for the polled trait in Nelore cattle were provided by ANCP, whereas Brahman data were collected at Fazenda Regalito (Flores de Goiás, Goiás, Brazil), Estâncias Espiritú (Beni, Bolivia), and Fazenda Guaporé Pecuária (Marca OB, Pontes e Lacerda, Mato Grosso, Brazil). The pedigree included 15 697 Brahman and 112 125 Nelore animals provided by ANCP.

Phenotyping of Brahman cattle was performed by a trained technician on animals with average age of 27.00 ± 22.69 months, using individual animal restraint to ensure both accuracy of data collected and safety of operators. The Brahman cattle distribution by birth year is presented in Figure 1. A standardized methodology was adopted, considering four phenotypic categories: polled (complete absence of horns), scab (thickened skin or small cornified protrusion without attachment to the skull), scur (horn‐like rudiment without attachment to the skull, up to 15 cm in length), and horned (horns firmly attached to the skull), as illustrated in Figure 2. The distribution of these phenotypes in Brahman cattle is presented in Table 1.

**FIGURE 1:**
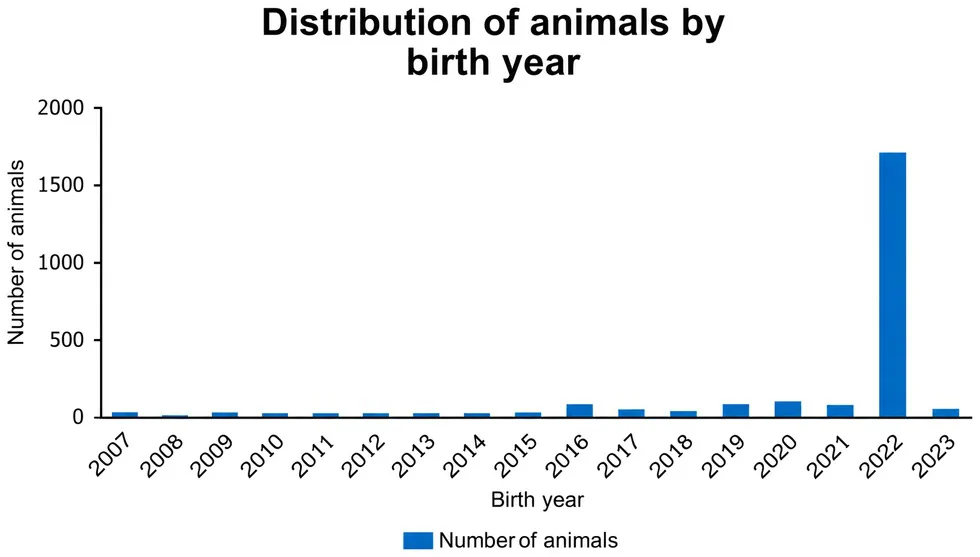
Distribution of the evaluated Brahman cattle by birth year.

**FIGURE 2:**
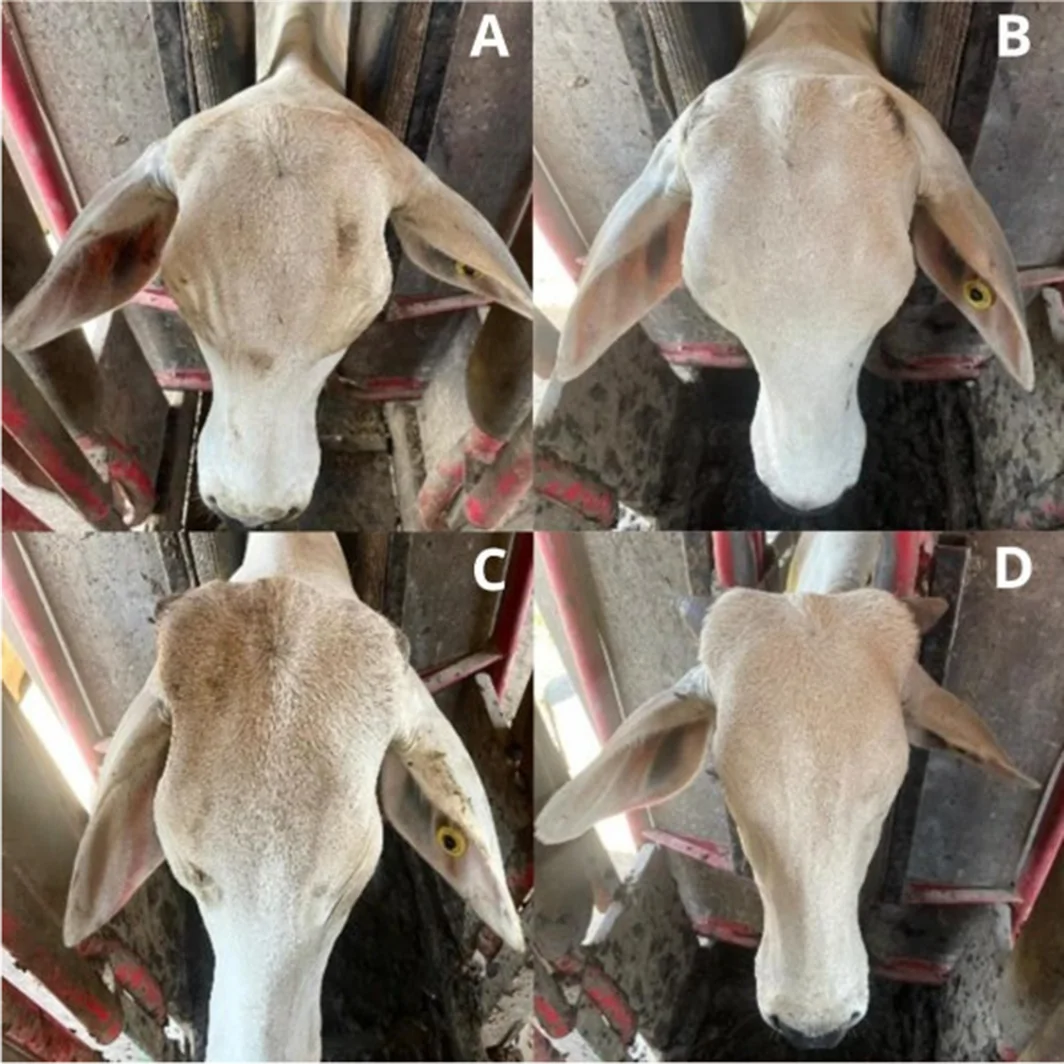
Shows the four phenotypic categories used to classify horn status in Brahman cattle: (A) Polled, (B) Scab, (C) Scur, and (D) Horned (horns attached to the skull) Brahman cattle.

**TABLE 1:** Percentage distribution of horn status in Brahman cattle, according to phenotypic categories observed at Fazenda Regalito, Estâncias Espiritú, and Fazenda Guaporé.

Category	Total (%)	Males (%)	Females (%)
Polled	5.06	0.37	8.32
Scab	14.86	19.66	11.52
Scur	23.26	24.16	22.63
Horned	56.82	55.81	57.53

Additionally, phenotypic classifications proposed by Temp et al. (2024) for Nelore cattle were also applied, considering alternative phenotypes: polled, polled from horned sire (hornless animals descended from horned parents), scurs/scabs, and horned. The distribution of these alternative phenotypes is presented in Table 2.

**TABLE 2:** Percentage distribution of alternative phenotypes for polled trait in Brahman cattle from Fazenda Regalito, Estâncias Espiritú, and Fazenda Guaporé.

Category	Total (%)	Males (%)	Females (%)
Polled	4.76	0.36	7.81
Polled from horned sire	0.29	0.00	0.49
Scurs and scabs	38.13	43.84	34.19
Horned	56.82	55.80	57.51

A total of 14 333 animals were genotyped, including both Nelore and Brahman cattle, with 53 492 SNPs initially available. Quality control was based on marker position, minor allele frequency (MAF), call rate, Hardy–Weinberg equilibrium (Fisher's exact test), and presence of monomorphic SNPs. Parent–offspring Mendelian conflicts were also assessed. SNPs were removed if they had call rate < 0.90, MAF < 0.05, were located on sex chromosomes (except in single‐breed models 3, 7, and 11, and in multi‐breed models), were monomorphic, had unknown positions in the ARS‐UCD1.2 reference genome, or presented Mendelian conflicts. After quality control, 14 333 animals and 45 790 informative SNPs (43 578 autosomal and 2212 non‐autosomal) were retained for analysis.

### 2.2 Evaluated Models, Genotyping Process, and Quality Control Analysis

To estimate variance components and genomic prediction accuracy for horn development in Brahman cattle using single‐breed models, 12 models with different phenotypic classifications were tested (Table 3).

**TABLE 3:** Evaluated models for genomic prediction of horn status in Brahman cattle (single‐breed analyses).

Models	Phenotype classification	Effects within CG			Sex outside CG	SNPs on sex chromosomes
		Farm	Year of birth	Sex		
1	Four observed phenotypes (polled, scab, scur, horned)	X	X	X		
2	Four observed phenotypes (polled, scab, scur, horned)	X	X		X	
3	Four observed phenotypes (polled, scab, scur, horned)	X	X			X
4	Four observed phenotypes (polled, scab, scur, horned)	X	X			
5	Two phenotypic classes (polled, scab, and scur vs. horned)	X	X	X		
6	Two phenotypic classes (polled, scab, and scur vs. horned)	X	X		X	
7	Two phenotypic classes (polled, scab, and scur vs. horned)	X	X			X
8	Two phenotypic classes (polled, scab, and scur vs. horned)	X	X			
9	Three phenotypic classes (polled, combined scab‐and‐scur, and horned)	X	X	X		
10	Three phenotypic classes (polled, combined scab‐and‐scur, and horned)	X	X		X	
11	Three phenotypic classes (polled, combined scab‐and‐scur, and horned)	X	X			X
12	Three phenotypic classes (polled, combined scab‐and‐scur, and horned)	X	X			

Abbreviation: CG, contemporary group.

Contemporary groups (CG) included herd and year of birth in all models. Sex was modeled differently: within CG (Models 1, 5, and 9), as a fixed effect outside CG (Models 2, 6, and 10), through SNPs on sex chromosomes (Models 3, 7, and 11), or excluded (Models 4, 8, and 12). Age was fitted as a linear covariate in all models.

Five additional models (13–22) were evaluated in the multi‐breed analyses (Table 4). In all models, herd and year of birth were included in the contemporary group (CG), while SNPs on sex chromosomes were modeled separately. Threshold models without metafounders were applied in Models 13, 16, 18, and 21, whereas linear models without metafounders were used in Models 14, 17, 19, and 22. Models 15 and 20 were fitted using threshold models including two metafounders, one assigned to each breed. In multi‐breed analyses (Models 16, 17, 21, and 22), breed effects were modeled by constructing the genomic relationship matrix (G) using breed‐specific allele frequencies, which has been shown to improve prediction accuracy (Cesarani et al. 2022).

**TABLE 4:** Evaluated models for genomic prediction of horn status in Brahman cattle, considering multibreed analyses and metafounders (MFs).

Models	Phenotype classification	Linear model	Threshold model	MF	Breed
13	Four observed phenotypes (polled, scab, scur, horned)		X		
14	Four observed phenotypes (polled, scab, scur, horned)	X			
15	Four observed phenotypes (polled, scab, scur, horned)		X	X	
16	Four observed phenotypes (polled, scab, scur, horned)		X		X
17	Four observed phenotypes (polled, scab, scur, horned)	X			X
18	Alternative phenotypes (polled, polled from horned sire, scabs and scurs, horned)		X		
19	Alternative phenotypes (polled, polled from horned sire, scabs and scurs, horned)	X			
20	Alternative phenotypes (polled, polled from horned sire, scabs and scurs, horned)		X	X	
21	Alternative phenotypes (polled, polled from horned sire, scabs and scurs, horned)		X		X
22	Alternative phenotypes (polled, polled from horned sire, scabs and scurs, horned)	X			X

Abbreviation: MF, metafounders.

The metafounder approach followed Legarra et al. (2015), which assumes a finite set of gametes for each ancestral population. Unlike traditional population genetics assumptions of independence, the metafounder theory allows ancestral populations to be genetically connected (Junqueira et al. 2020). Therefore, according to the metafounder theory, ancestral populations may not be independent but rather connected through underlying genetic links. In this study, metafounders were defined according to the ancestral breed groups represented in the population under investigation. Specifically, one metafounder was assigned to the Nelore breed and another to the Brahman breed, which allowed the model to account for shared genetic ancestry between the two populations and to better capture historical relationships across breeds. Londoño‐Gil et al. (2024) performed a principal component analysis (PCA) of a multi‐breed population composed of Nelore, Brahman, Guzerá, and Tabapuã cattle, highlighting the historical genetic relationship between Brahman and Nelore populations.

These models were designed to identify the most appropriate phenotypic classification for horn development in Brahman cattle. Given the complex genetic architecture of the polled trait, they also tested different assumptions regarding inheritance patterns and prediction approaches, aiming to improve the accuracy of genomic predictions.

### 2.3 Estimation of Variance Components

For the estimation of variance components in single‐breed models, a threshold model with Bayesian approach was applied using the Gibbsf90+ software, available in the BLUPF90+ package (Misztal 2022). In the context of threshold models, it was assumed that the underlying scale follows a continuous normal distribution, represented as:

(1) $$u|\mathrm{\theta N}\left(\mathrm{W\theta},I\sigma_{e}^{2}\;\right)$$

where u is the vector of the underlying scale with order *r*; θ is the vector of location parameters; W is the incidence matrix of order *r × s*; I is an identity matrix of order *r × r*; and $\sigma_{e}^{2}$ is the residual variance. According to Van Tassell et al. (1998), Bayesian threshold models for categorical traits are particularly suitable for estimating variance components and breeding values, as they relate categorical responses to an unobservable continuous scale. For these models, the residual variance was fixed at 1.

Categorical traits were treated as continuous unobservable variables on the underlying scale, with fixed initial threshold values. Let t1 < t2… < t j‐1, with t0 = −∞ and tj = ∞, where *j* is the number of categories. The observed data are intrinsic to the unobservable variable, restricted between two thresholds (Gianola and FOulley 1983). Thus, the categories of *y*_*i*_ for each animal *i* were defined by *U*_*i*_ on the underlying scale:

(2) $$y_{i}=\left\{\begin{array}{c} \begin{array}{ccc} 1_{t0} & <U_{i} & \leq t_{1}\\ 2_{t1} & <U_{i} & \leq t_{2}\\ 3_{t2} & <U_{i} & \leq t_{3} \end{array}\\ 4_{t3}\;<U_{i}\;\leq t_{4}\; \end{array}\right.\;\begin{array}{c} 0\\ 0\\ \begin{array}{c} 0\\ i-1,\ldots ,n \end{array} \end{array}$$

where ***n*** is the number of observations. Following thresholds *t*_0_ to *t*_4_, one threshold (between *t*_1_ and *t*_3_) must be fixed to an arbitrary constant. In this analysis, *t*1 = 0 was assumed, defining the vector of estimable thresholds as follows:

(3) $$t=\left[\frac{t_{2}}{t_{3}}\right]$$

Gibb's sampling, a numerical integration procedure introduced by Geman and Geman (1984) and considered a specific case of the Metropolis–Hasting's algorithm, was applied. This method uses the full conditional posterior distributions of parameters, consistent with known probability density functions (Gelman et al. 2013). Chains of 200 000 cycles were generated, with burn‐ins of 50 000 and sampling intervals every 100 cycles. Posterior estimates of variance components in single‐breed models were obtained using POSTGIBBSF90, also available in the BLUPF90 package (Misztal et al. 2014).

Convergence was assessed by visual inspection of trace plots and posterior density plots of genetic and residual variances, as well as using the method proposed by Geweke (1992), implemented in the Bayesian Output Analysis (BOA) package (Smith 2005) in the R software (R Core Team 2023). According to this method, convergence is declared when no significant differences (*p* > 0.05) are detected between the initial and final segments of the Markov chain. Lack of convergence is indicated when *p*‐values are below 0.05. Additionally, autocorrelation coefficients among samples were examined. As observed by Cowles and Carlin (1996), autocorrelation analysis evaluates the independence of posterior samples; highly correlated values indicate strong sample dependence and reduced precision of estimates. For multi‐breed analyses, variance components obtained from the study of Temp et al. (2024) were adopted.

### 2.4 Genomic Prediction

Genomic prediction of the polled trait was performed using single‐breed threshold models (Models 1–12), multi‐breed threshold models (Models 13, 16, 18, and 21), and multi‐breed linear models (Models 14, 15, 17, 19, 20, and 22). In Models 16, 17, 21, and 22, the breed effect was included. In Models 15 and 20, metafounders were incorporated. Analyses were carried out using the single‐step genomic best linear unbiased predictor (ssGBLUP) approach. Linear models were fitted with blup90iod3, while threshold models were implemented using cblup90iod2OMP, both available in the BLUPF90+ package (Misztal 2022).

In ssGBLUP, the pedigree‐based relationship matrix is adjusted with the genomic relationship matrix for genotyped animals. According to Aguilar et al. (2010), the inverse of the additive relationship matrix (*A* ^−1^) can be represented by the inverse of the *H* matrix (𝐻^−1^) as:

(4) $$H^{-1}=A^{-1}+\left[\begin{array}{cc} 0 & 0\\ 0 & G^{-1}-A_{22}^{-1} \end{array}\right]$$

where *H* ^−1^ is the inverse relationship matrix combining animals with phenotypes, genotypes, or both; *A* ^−1^ is the inverse of the pedigree‐based relationship matrix for all animals; $A_{22}^{-1}$ is the inverse pedigree‐based relationship matrix for genotyped animals; and *G* ^−1^ is the inverse genomic relationship matrix.

For multi‐breed analyses with metafounders, the inverse relationship matrix including pedigree, genomic, and metafounder information (*H* ^Γ−1^) was calculated using blup90iod3 (Lourenco et al. 2022; Misztal et al. 2014). In this context, ssGBLUP is expressed as follows:

(5) $$H^{\Gamma -1}=A^{\Gamma -1}+\left[\begin{array}{cc} 0 & 0\\ 0 & G^{-1}-A_{22}^{\Gamma -1} \end{array}\right]$$

where *H* ^Γ−1^ is the inverse matrix combining pedigree, genomic, and metafounder information; *A* ^Γ−1^ is the inverse additive relationship matrix with metafounders; *G* ^Γ−1^05 is the inverse genomic relationship matrix for genotyped animals including metafounders; and $A_{22}^{\Gamma -1}$ is the additive relationship matrix among genotyped animals with metafounders.

In this study, genomic estimated breeding values (GEBVs) were transformed to the probability scale using the *pnorm* function in the R software (R Core Team 2023). These values were expressed as expected progeny differences (EPDs), representing the probability of an individual transmitting the polled phenotype to its offspring compared with the population average (ANCP 2024).

### 2.5 Prediction Accuracy, Bias, and Dispersion

To evaluate the accuracy of genomic estimated breeding values (GEBVs) for the polled trait, data subsets were created following the LR method (linear regression) proposed by Legarra and Reverter (2018). Two subsets were considered: whole (w) and partial (p).

The whole subset included genotyped animals with or without phenotypes but with progeny information, non‐genotyped animals with phenotypes, and genotyped animals with phenotypes but no progeny information. The partial subset consisted of young, genotyped animals born in 2021, 2022, and 2023 (*n* = 222). In this subset, phenotypic information was masked. This validation strategy simulates practical genomic selection, in which the prediction of GEBVs for the selection of young candidates is based on the phenotypic and genotypic information of proven and older animals in the complete dataset.

The LR method was preferred over conventional cross‐validation because it provides simultaneous estimates of prediction accuracy, bias, and dispersion, while making more efficient use of the available data in relatively small reference populations, thereby avoiding the reduction in training population size inherent to cross‐validation (Legarra and Reverter 2018).

Prediction accuracy was calculated as described by Hill (2010):

(6) $$\mathrm{acc}=\frac{\mathrm{cov}}{\sqrt{\left(1-F\right).\mathrm{var}\left(a\right)}}$$

where acc is the prediction accuracy; cov is the covariance between true and predicted breeding values; *F* is the average inbreeding coefficient; var(*a*) is the additive genetic variance; and *a* is the additive genetic value.

Bias was estimated as the mean difference in GEBVs between the partial and whole datasets, following Legarra and Reverter (2018):

(7) $$\mu_{w,p}=\bar{{û}_{p}}-\bar{{û}_{w}}$$

where $\mu_{w,p}$ is the bias, defined as the difference between the mean GEBVs estimated from the partial and whole datasets; $\bar{{û}_{p}}$ is the mean GEBV estimated using the partial dataset; and $\bar{{û}_{w}}$ is the mean GEBV estimated using the whole dataset.

Dispersion was calculated as the slope of the regression of GEBVs from the whole dataset on those from the partial dataset, also according to Legarra and Reverter (2018):

(8) $$b_{w,p}=\frac{\mathrm{cov}\;\left({û}_{p}-{û}_{w}\right)}{\mathrm{var}\;{û}_{p}}$$

where $b_{w,p}$ is the dispersion, defined as the regression coefficient (slope) of GEBVs estimated from the whole dataset against GEBVs estimated from the partial dataset; $\mathrm{cov}\;\left({û}_{p}-{û}_{w}\right)$ is the covariance between GEBVs estimated from the partial and whole datasets; and $\mathrm{var}\;{û}_{p}$ is the variance of GEBVs estimated from the partial dataset.

As observed by Jang et al. (2022), prediction accuracy, bias, and dispersion are the main efficiency indicators in genomic prediction models. Macedo, Christensen, et al. (2020) further reported that these parameters are effective in assessing the predictability of the selection response, model correction, and the contribution of variance components and effects not explicitly included in the models.

In addition, the Spearman's rank correlation coefficient was computed for each model after ranking animals according to their predicted breeding values, comparing results between the whole and partial datasets. This parameter quantifies the consistency of association between the two ranked variables.

The inbreeding coefficient was calculated using INBUPGF90, available in the BLUPF90+ package. The mean inbreeding coefficient estimated for the partial subset was 0.01997.

## 3 Results and Discussion

### 3.1 Single‐Breed Analyses

The posterior estimates of variance components for direct additive genetic and residual effects, as well as estimates of heritability (*h* ^2^), standard deviations, confidence intervals, prediction accuracy, bias, and dispersion obtained from the single‐breed analyses are presented in Table 5.

**TABLE 5:** Posterior estimates of variance components and prediction ability for horn development in Brahman cattle using different single‐breed models.

Posterior density estimates for variance components						Prediction accuracy		
Model	*σ* ^2^a	*σ* ^2^e	*h* ^2^	±SD	CI (95%)	ρ_*p,w*_	*μ*_*w,p*_	*b*_*w,p*_
1	1.53	1.12	0.57	0.05	0.4594–0.6777	0.53	0.13	0.74
2	1.52	1.12	0.57	0.05	0.4640–0.6831	0.41	0.17	1.17
3	1.49	1.49	0.56	0.06	0.4426–0.6728	0.53	0.13	0.78
4	1.54	1.12	0.57	0.05	0.4568–0.6830	0.55	0.12	0.64
5	3.08	1.00	0.72	0.07	0.5830–0.8809	0.39	0.20	−0.03
6	3.04	1.00	0.72	0.07	0.5700–0.8699	0.45	0.14	0.94
7	2.90	1.00	0.71	0.07	0.5764–0.8620	0.45	0.11	0.97
8	3.06	1.00	0.72	0.07	0.5861–0.8678	0.47	0.04	0.77
9	1.52	0.91	0.60	0.07	0.4668–0.7549	0.56	0.09	0.70
10	1.48	0.91	0.60	0.07	0.4641–0.7500	0.52	0.10	0.80
11	1.35	0.91	0.58	0.07	0.4459–0.7244	0.53	0.10	0.85
12	1.48	0.91	0.60	0.07	0.4600–0.7395	0.56	0.10	0.68

Abbreviations: *b*_*w*,*p*_, dispersion; CI, confidence interval; h^2^, heritability; *μ*_*w*,*p*_, bias; *ρ*_*p*,w_, prediction accuracy; *σ* ^2^a, additive genetic variance; *σ* ^2^e, residual variance; ±SD, standard deviation.

In this study, higher *h* ^2^ estimates were observed in single‐breed models with two phenotypic classifications (Models 5, 6, 7, and 8), while lower estimates were obtained in models with four observed phenotypic categories (Models 1, 2, 3, and 4). Overall, within each phenotypic classification, *h* ^2^ estimates were similar regardless of model structure or inclusion of specific effects.

The relatively high estimates suggest that horn development in Brahman cattle exhibits sufficient additive genetic variability, indicating that it may be responsive to selection. Consequently, selection could yield genetic gain in subsequent generations. Moreover, horn status could serve as a complementary selection criterion for breeders seeking to increase the frequency of naturally polled animals in their herds.

The confidence intervals of *h* ^2^ estimates from single‐breed models were relatively narrow, indicating good precision and low uncertainty. In the present single‐breed analyses, alternative phenotypes were not considered. This decision was based on the observed data distribution, which differs from that reported for Nelore cattle. In Nelore cattle, a large number of polled females are typically mated with horned males to increase the frequency of polled offspring. In Brahman cattle, however, naturally polled females are rare at the population level, making such practices uncommon. Thus, the use of alternative phenotypes does not appear appropriate or feasible when considering Brahman cattle alone.

For Nelore cattle, Temp et al. (2024) reported *h* ^2^ estimates of 0.58–0.60 when using alternative phenotypes. In comparison, these authors found lower *h* ^2^ estimates (0.44–0.45) with two‐category models and higher estimates (0.83–0.84) with three‐category models. Although these high estimates suggest strong genetic control, they may also reflect misclassification of true phenotypes, resulting in upward bias.

Like horns, intermediate phenotypes such as scabs and scurs—identified in this study as non‐fixed cranial bone structures with variable size, shape, and length—are likely influenced by multiple genes and vary considerably between breeds and sexes (Mariasegaram et al. 2012). Wiedemar et al. (2014) further reported that these phenotypes are heterozygous for the polled locus.

Gehrke et al. (2020), analyzing Holstein‐Friesian data, observed that the Friesian variant exhibited stronger epistatic effects on scabs and scurs compared with the Celtic variant. They also observed that the expression of these structures depends on age at phenotyping, sex, and breed. Randhawa et al. (2020) emphasized the complexity of scoring scabs, scurs, and horns in young animals, as horn buds initially present unstable attachments before consolidating into true horns. Grobler et al. (2021) reported that horn bud development begins within the first 2 months of life, reaching full penetrance by 6 months.

In the present study, phenotyping was performed in animals averaging 27 months (±22.69), ensuring reliable classification of observed phenotypes. Females were more represented in the polled and horned categories compared with males. This supports the hypothesis that horn development differs by sex, where horn, scab, and scur phenotypes are thought to be dominant in males, while the polled phenotype is dominant in females (Mariasegaram et al. 2012; Stafuzza et al. 2018; Gehrke et al. 2020; Simon et al. 2022). Our results, however, diverged from the expectation of higher horn frequency in males, as more horned females were recorded. Nevertheless, the observation of more polled females than males agrees with the reported inheritance pattern.

For *Bos indicus* cattle, the exact locus associated with scabs and scurs has not yet been identified. Multiple genes are presumed to influence their expression. Mariasegaram et al. (2012) highlighted the need for standardized protocols to accurately distinguish polled, scab, scur, and horned phenotypes.

From a broader perspective, prediction accuracy, bias, and dispersion estimates for the polled trait in Brahman cattle were generally unfavorable in single‐breed analyses (Table 5). Particularly, Models 2 and 5 showed low prediction accuracies (0.41 and 0.39), high biases (0.17 and 0.20), and unsatisfactory dispersion (1.17 and −0.03, respectively). The lack of robustness in single‐breed analyses can be explained by the relatively small population size of Brahman cattle. Consistent with this, Wicki et al. (2023) observed that effective population size significantly impacts the reliability of genomic evaluations. Similarly, Cesarani et al. (2022) reported that reference population size strongly influences the accuracy of genomic predictions.

Legarra and Reverter (2018) indicated that bias 𝜇_𝑤,𝑝_ = *û¯*_𝑝_– *û¯*_𝑤_ is expected to approach zero in unbiased predictions, while dispersion (bw,*p*) should approach 1 when estimates are neither inflated nor deflated.

### 3.2 Multi‐Breed Analyses

Among the models evaluated, Model 20 (alternative phenotypes, multi‐breed, including metafounders, sex effect within CG, and sex‐chromosome SNPs) showed the highest prediction accuracy, reduced bias, and dispersion close to 1 (Table 6). These findings validate the hypothesis that multi‐breed populations and metafounders improve predictive efficiency for the polled trait, making them essential for robust genomic evaluations with lower prediction errors.

**TABLE 6:** Estimates of accuracy, bias, and dispersion for horn development in Brahman cattle using different multibreed models.

Model	ρ_*p,w*_	*μ*_*w,p*_	*b*_*w,p*_
13	0.57	1.07	0.80
14	0.62	0.82	0.89
15	0.74	0.38	1.02
16	0.64	0.38	0.77
17	0.63	0.24	0.79
18	0.59	0.83	0.78
19	0.66	0.77	1.00
20	0.79	0.36	1.06
21	0.71	0.38	0.80
22	0.78	0.23	0.80

Abbreviations: 𝑏_𝑤_, dispersion; 𝜇_𝑤_, bias; 𝜌_𝑝_, prediction accuracy.

The use of multi‐breed populations with metafounders increased the number of animals available for analysis and improved compatibility between genomic and pedigree information (Meyer and Swan 2019). This is expected given the genetic contribution of Nelore to Brahman cattle (Koufariotis et al. 2018) and the smaller sample size and fewer phenotypic and genotypic records available for Brahman cattle. As a result, improvements of ~7% in prediction accuracy were observed when using observed phenotypes (Model 3), ~39% with observed phenotypes plus metafounders (Model 15), and ~49% with alternative phenotypes, metafounders, and multi‐breed analysis (Model 20).

Comparable gains in prediction reliability have been reported in cattle (Hayes et al. 2019; Van Den Berg et al. 2019; Khansefid et al. 2020), swine (Yin et al. 2024), and sheep (Wicki et al. 2023) through combining populations and applying metafounders. Similarly, Londoño‐Gil et al. (2024) found that multi‐breed models with metafounders, although sometimes biased, achieved higher accuracy and lower dispersion, particularly benefiting breeds with smaller population sizes.

In our study, the multi‐breed model with observed phenotypes and metafounders (Model 15) yielded accuracy, bias, and dispersion estimates of 0.74, 0.38, and 1.02, respectively. Although less favorable than Model 20, this difference likely reflects misclassification of heterozygous animals when using observed phenotypes (polled, scab, scur, horned) since homozygous and heterozygous polled animals are grouped into the same category.

As Mariasegaram et al. (2012) pointed out, phenotypic selection against horns remains challenging due to the dominant inheritance of the polled trait. Homozygous and heterozygous polled animals cannot be phenotypically distinguished, complicating selection strategies. Thus, accurate phenotyping and appropriate modeling are essential for improving genetic evaluations and understanding the biological basis of the polled trait.

Our findings are consistent with studies showing that metafounders improve prediction ability (Garcia‐Baccino et al. 2017; Macedo, Reverter, and Legarra 2020). Kluska et al. (2021) emphasized that in synthetic or composite breeds such as Brahman, incompatibilities between genomic (G) and pedigree (A) matrices can be exacerbated by population heterogeneity, but these issues are mitigated by metafounders (Legarra et al. 2015). Indeed, metafounders improve the compatibility between A and G by modeling shared ancestry (Kudinov et al. 2020).

Londoño‐Gil et al. (2024) reported metafounder relationships of 0.69 within Brahman cattle and 0.64 between Nelore cattle and Brahman cattle, demonstrating that base populations within and across breeds are genetically related, reflecting ancestral connections.

Finally, one strategy adopted here was the inclusion of breed effects in multi‐breed models. As suggested by Lourenco et al. (2016), this approach improves the compatibility between A and G matrices by incorporating breed‐specific allele frequencies. Models 16 and 17 (observed phenotypes with breed effect) showed similar accuracy (0.64 and 0.63) and dispersion (0.77 and 0.79), with Model 17 showing lower bias. Similarly, Models 21 and 22 (alternative phenotypes with breed effect) achieved higher accuracies (0.71 and 0.78) and lower biases (0.38 and 0.23), with dispersion of 0.80 in both cases.

Although the polled trait is inherently a binary trait (presence vs. absence of horns), both threshold and linear models were evaluated. Differences between Models 16/17 and 21/22 reflect not only the phenotypic classification (observed vs. alternative) but also the modeling approach (threshold vs. linear). Linear models 17 and 22 yielded lower bias than their threshold counterparts. Similar findings have been reported in cattle (Kizilkaya et al. 2014; Campos et al. 2019) and swine (Cappelloni et al. 2022; Hidalgo et al. 2024), highlighting that linear models may perform equally well as threshold models for categorical traits while offering computational simplicity (Mrode 2005).

Despite the relatively small population size of the Brahman cattle in Brazil (Londoño‐Gil et al. 2024), the multi‐breed model with metafounders and alternative phenotypes (Model 20) provided the best predictive performance. The alternative classification (1. polled; 2. polled from horned sire; 3. scabs and scurs; 4. horned) more comprehensively represented horn‐related phenotypes in Brahman cattle. The superior performance of Model 20 is likely explained by the combination of a biologically more informative phenotypic classification and the inclusion of metafounders, which account for the shared ancestry between Brahman cattle and Nelore cattle. Together, these features improved the representation of genetic relationships among animals and increased the accuracy of genomic predictions. Therefore, this study demonstrates that the appropriate choice of the statistical model and phenotypic classification strategy is essential for improving the performance of genomic evaluations for horn development in Brahman cattle. Additionally, based on these results, it is possible to propose a new expected progeny difference (EPD) for the polled trait in Brahman cattle, providing a practical selection tool for breeders. Incorporating this trait into breeding programs could enhance the productive, economic, and sustainable potential of beef cattle production.

## 4 Conclusion

The high heritability estimates obtained in this study indicate that the absence of horns in Brahman cattle is a trait amenable to selection and, when combined with genomic information, represents a viable alternative to permanently reduce the need for surgical dehorning and thereby improve animal welfare. Single‐breed models using observed phenotypic categories were not suitable for the accurate genomic prediction of this trait.

Incorporating multi‐breed analyses and metafounders substantially improved the predictive ability for the polled trait in Brahman cattle, proving highly promising for the study of complex traits in breeds with small population sizes. The results demonstrate strong potential for increasing the frequency of alleles associated with the polled phenotype in future generations of Brahman cattle.

However, it is important to highlight that, although high heritability estimates were obtained, increasing the number of phenotypic and genotypic records in the population under study may further improve prediction accuracy. This study represents an initial contribution toward a better understanding of the genetic architecture of the polled trait in multi‐breed Brahman cattle populations. Future investigations involving larger datasets of phenotyped and genotyped animals, as well as more balanced phenotype distributions, are expected to provide more reliable genomic predictions and further support the implementation of genomic selection for this trait.

## Author Contributions

**Lanna Maryana Costa Pereira:** conceptualization, data management, methodology, statistical analysis, investigation, writing – original draft, writing – review and editing. **Ludmilla Costa Brunes:** conceptualization, methodology, statistical analysis, investigation, writing – original draft, writing – review and editing. **Cláudio Ulhôa Magnabosco:** conceptualization, data management, resources, methodology, funding acquisition. **Fernando Baldi:** conceptualization, data curation, methodology, statistical analysis, investigation, writing – original draft, writing – review and editing. **Luís Camaripano:** data management.

## Funding

This work was supported by Coordenação de Aperfeiçoamento de Pessoal de Nível Superior (001).

## Ethics Statement

This study, registered under protocol no. 76‐24, involving the production, maintenance, and/or use of animals belonging to the phylum Chordata, subphylum Vertebrata (except humans) for scientific research (or teaching), complies with the requirements of Law no. 11.794 (October 8, 2008), Decree no. 6.899 (July 15, 2009), and the guidelines issued by the National Council for the Control of Animal Experimentation (CONCEA). It was approved by the Ethics Committee on Animal Use (CEUA) of the Federal University of Goiás (UFG) on September 23, 2024.

## Conflicts of Interest

The authors declare no conflicts of interest.

## Data Availability

The data supporting the findings of this study can be accessed under a Material Transfer Agreement, subject to confidentiality restrictions. These data are not publicly available due to legal restrictions, as they belong to a commercial breeding program.
